# A Brain-Computer Interface Based Cognitive Training System for Healthy Elderly: A Randomized Control Pilot Study for Usability and Preliminary Efficacy

**DOI:** 10.1371/journal.pone.0079419

**Published:** 2013-11-18

**Authors:** Tih-Shih Lee, Siau Juinn Alexa Goh, Shin Yi Quek, Rachel Phillips, Cuntai Guan, Yin Bun Cheung, Lei Feng, Stephanie Sze Wei Teng, Chuan Chu Wang, Zheng Yang Chin, Haihong Zhang, Tze Pin Ng, Jimmy Lee, Richard Keefe, K. Ranga Rama Krishnan

**Affiliations:** 1 Duke-National University of Singapore, Graduate Medical School, Singapore, Singapore; 2 Singapore Clinical Research Institute, Singapore, Singapore; 3 Institute for Infocomm Research, Agency for Science, Technology and Research, Singapore, Singapore; 4 National University of Singapore, Singapore; 5 Institute of Mental Health, Singapore; 6 Duke University, Durham, North Carolina, United States of America; University of Glasgow, United Kingdom

## Abstract

Cognitive decline in aging is a pressing issue associated with significant healthcare costs and deterioration in quality of life. Previously, we reported the successful use of a novel brain-computer interface (BCI) training system in improving symptoms of attention deficit hyperactivity disorder. Here, we examine the feasibility of the BCI system with a new game that incorporates memory training in improving memory and attention in a pilot sample of healthy elderly. This study investigates the safety, usability and acceptability of our BCI system to elderly, and obtains an efficacy estimate to warrant a phase III trial. Thirty-one healthy elderly were randomized into intervention (n = 15) and waitlist control arms (n = 16). Intervention consisted of an 8-week training comprising 24 half-hour sessions. A usability and acceptability questionnaire was administered at the end of training. Safety was investigated by querying users about adverse events after every session. Efficacy of the system was measured by the change of total score from the Repeatable Battery for the Assessment of Neuropsychological Status (RBANS) before and after training. Feedback on the usability and acceptability questionnaire was positive. No adverse events were reported for all participants across all sessions. Though the median difference in the RBANS change scores between arms was not statistically significant, an effect size of 0.6SD was obtained, which reflects potential clinical utility according to Simon’s randomized phase II trial design. Pooled data from both arms also showed that the median change in total scores pre and post-training was statistically significant (Mdn = 4.0; *p*<0.001). Specifically, there were significant improvements in immediate memory (p = 0.038), visuospatial/constructional (p = 0.014), attention (p = 0.039), and delayed memory (p<0.001) scores. Our BCI-based system shows promise in improving memory and attention in healthy elderly, and appears to be safe, user-friendly and acceptable to senior users. Given the efficacy signal, a phase III trial is warranted.

**Trial Registration:**

ClinicalTrials.gov NCT01661894

## Introduction

By 2050, the proportion of people aged 60 years and above is estimated to reach 22% of the world population [1]. Cognitive decline is one of the most pervasive consequences of aging, and is associated with loss of autonomy, functional impairment and deterioration in quality of life [2]. From an economic perspective, cognitive decline contributes to healthcare expenditures that are almost tenfold higher for individuals with such deficits than those without [3]. Clearly, there is an urgent need to develop empirically validated interventions for preserving the cognitive functions in the elderly.

In recent years, there has been growing interest in the use of computer-based cognitive training programs for this purpose. Several studies have demonstrated that cognitive training can improve cognitive functioning in various domains such as memory [4]–[7], attention [8]–[9], processing speed [10]–[12] and executive functioning [11], [12] in elderly populations, and that such gains can result in better functional outcomes even up to five years after training [13]. Meta-analyses examining the efficacy of both traditional cognitive interventions [14] and computer-based training programs in healthy elderly populations have found similar positive results [15]. While traditional cognitive interventions often involve an extensive amount of face-to-face contact with trained instructors [15], computer-based interventions are low-cost and can be implemented anywhere, which is particularly important for elderly who may have mobility and financial issues. They are also often designed to be enjoyable, which aids in motivating users to adhere to the training regimen.

Brain-computer interface (BCI) is a communication method based on brain neural activity [16]. It has generally been used in motor rehabilitation after stroke or spinal injuries (e.g. [17], [18]). However, there has been recent interest in its potential for improving cognitive function, especially as an alternative to neurofeedback training (NFT). NFT is an operant-conditioning protocol that trains individuals by providing them with real-time visual or auditory feedback about their electroencephalographic (EEG) brainwave patterns [19]. While NFT offers real-time, personalized feedback, the task involved is typically repetitive and monotonous in nature, as its sole purpose is to indicate to participants if they have achieved the optimal brainwave pattern. In addition, NFT requires individuals to wear an electrode cap throughout training, which is inconvenient, increases preparation time and may lead to discomfort for users. There are also scant few studies examining NFT in elderly populations [20], [21].

In 2010, our team developed a novel EEG-based BCI training program that combines the advantages of traditional computerized training interventions and NFT. It offers real-time, individualized training with BCI, coupled with the relatively more engaging game interfaces of computerized training interventions. Our BCI system quantifies one’s attention level via EEG waves, which are recorded by two dry electrodes on a headband, corresponding roughly to the frontal (Fp1, Fp2) positions. EEG signals are transmitted via Bluetooth to the computer. The system passes incoming EEG signals through a filter bank and common spatial pattern filtering to determine attentive states, which are translated into quantifiable scores that allow a user to control a computer game using his attention. The system was calibrated for each user using a Stroop task before training. In previous studies, our device has shown promise as a novel treatment for improving attention in children with ADHD, by improving parent-rated inattentive scores on the ADHD Rating Scale [22], [23]. It must be noted that while our previous studies were conducted on a different population from the current one (children with ADHD vs healthy early population), our BCI system focuses on the modality of attention, and intervention effects are not expected to be specific to a particular population segment per se. Whereas the training program used in our previous studies for children with ADHD focused solely on improving attention, we modified the BCI system using a new game with a memory training component targeted for the elderly population in this current study.

In the present paper, we thus examine the feasibility of using our BCI-based system in improving memory and attention in a pilot sample of healthy elderly. This study aimed to:

- Determine the usability and acceptability of the BCI device for the elderly participants;
- Assess study adherence and identify any safety concerns;
- Obtain an estimate of efficacy in improving memory and attention in healthy elderly participants to determine whether the study should proceed to a phase III trial.

## Methods

The protocol for this trial and supporting CONSORT checklist are available as supporting information; see Checklist S1 and Protocol S1.

### Ethics Statement

This study was approved by the Institutional Review Board of the National University of Singapore. Written informed consent was obtained prior to study entry (Clinicaltrials.gov registration no. NCT01661894). There was a slight delay in trial registration due to administrative oversight.

### Study Design

**Figure 1:**
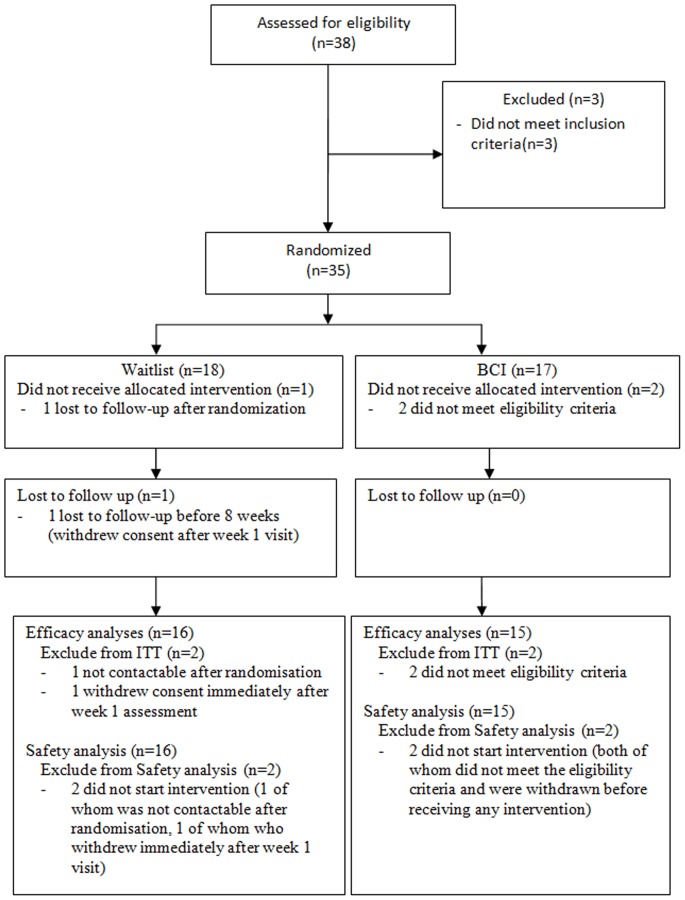
CONSORT flow diagram.

This was a two-arm, randomized, wait-list control trial to assess BCI as a means of improving attention and memory in healthy elderly. The Consort Flow Diagram is shown in Figure 1. Simon’s randomized phase II design was used for determining if the BCI system was worthy of further evaluation [24]. Patients were randomized into either BCI intervention group or waitlist control group via direct web randomization. Randomization was done in a 1∶1 allocation ratio, stratified by education level. Authorized study centre personnel randomized the participants via a password protected internet website. The randomization system determined the treatment and provided a randomization number to be used for each participant. Blocking was used in the randomization process. The block length was determined by a biostatistician but is not revealed to the clinical team as per ICH E9 guidelines. Potential participants were recruited by the following methods: (a) from the Singapore Longitudinal Aging Study (SLAS), a large-scale cohort study of elders in Singapore [25]; (b) by approaching the organizers of relevant seminars for permission to recruit during the events; (c) by word-of-mouth from recruited subjects. Participants were deemed eligible for the study if they met all of the following criteria at pre-screening: aged between 60–70 years old, Geriatric Depression Scale (GDS) of 9 and below, Mini-Mental State Examination of 26 and above, Chinese ethnicity, literate in English, able to travel to study site independently, absence of known neuropsychiatric disorders (such as epilepsy or mental retardation), and were not participating in another research study (aside from the SLAS).

### Procedure

The BCI system used in the present study has been elaborated upon in a previous publication [23]. Before training, all participants underwent a calibration Stroop task. This Stroop task allowed the BCI system to develop an individualized EEG profile representing each participant’s attentive state [23].

**Figure 2:**
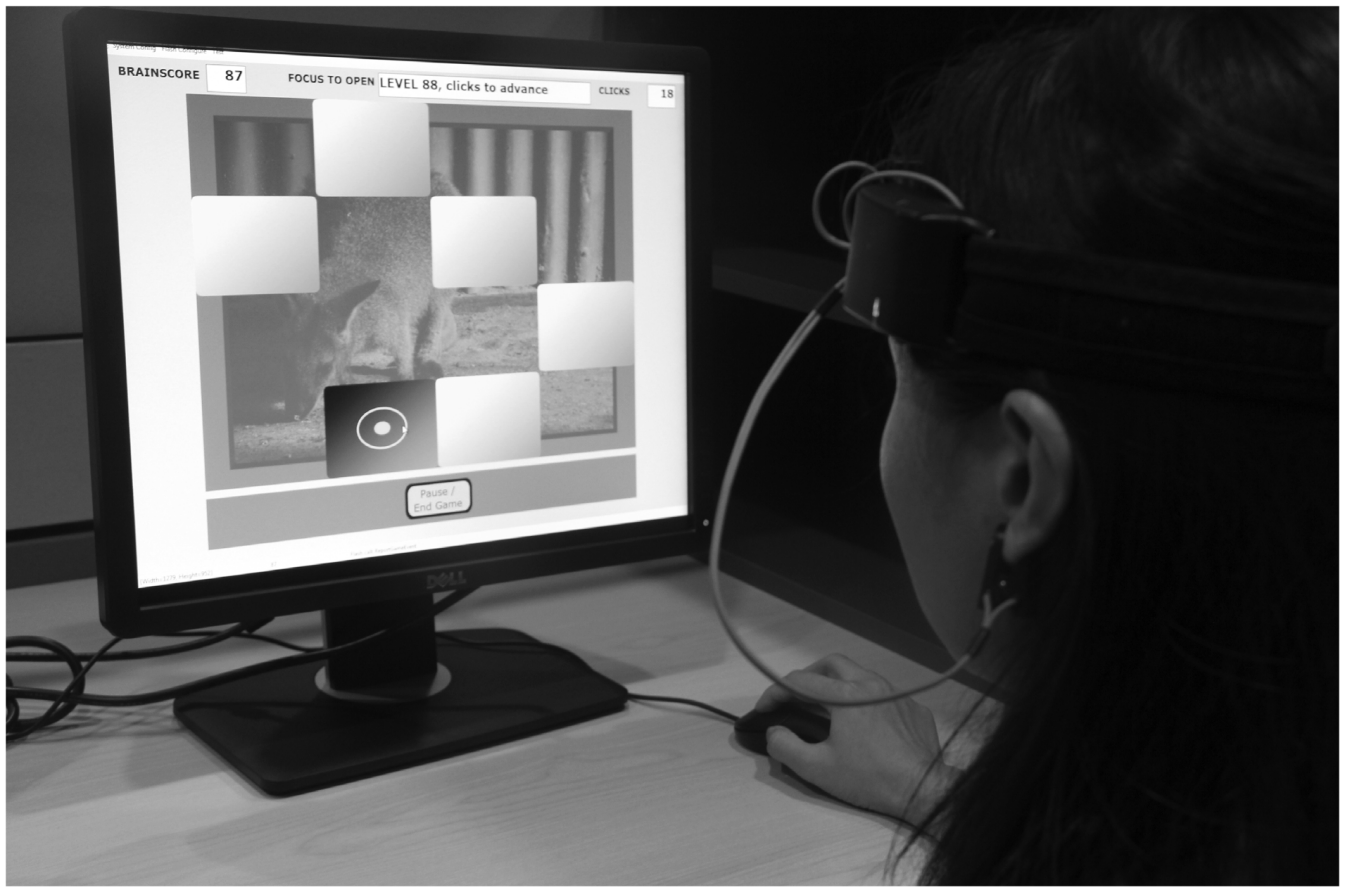
A model engaged in the Brain Computer Interface (BCI) memory and attention training game system. The model has given written informed consent, as outlined in the PLOS consent form, to publication of her photograph.

Participants in both the intervention and the wait-list group underwent the BCI intervention for 24 sessions over the span of 8 weeks. Each session was planned to take 30-minutes to complete. During each session, participants played a card-pairing memory game, in which they had to focus their attention in order to open or close the cards on screen (see Figure 2). After each training session, participants were queried as to whether they experienced any adverse events.The intervention group underwent the BCI treatment in their first 8 weeks of being in the trial. The waitlist control arm did not undergo the intervention until after 8 weeks. From Week 9 to 16, the waitlist control arm underwent the same BCI training intervention procedure as the Intervention Arm.

The Repeatable Battery for the Assessment of Neuropsychological Status (RBANS) was administered at three time points for each participant. It was administered before the start of BCI training at Week 1, immediate post training (Week 8) and two months post training (Week 16) for intervention group. The waitlist control group on the other hand, did RBANS at Week 1 and Week 9 before BCI training, and Week 16 immediately post training.

All study procedures from the first recruitment to the last follow up was completed between April 2012 and January 2013.

### Outcome Measures

A usability and acceptability questionnaire (adapted from [26]) was administered at each participant’s final BCI training session (week 8 for intervention arm and week 16 for the waitlist control). Participants rated how strongly they agreed with each item on a scale of 1(Strongly Disagree) to 7 (Strongly Agree) (see Table 1).

**Table 1:** Descriptive summary of responses to all items in the usability and acceptability questionnaire.

Questionnaire item	Mean	SD	Median	Range
1. Overall I am satisfied with how easy it is to use this device.	6.4	0.8	7	4 to 7
2. I feel comfortable using this device.	6.4	0.7	6	5 to 7
3. I enjoyed playing the game.	6.8	0.5	7	5 to 7
4. I think the device is useful in training my memory and attention.	6.6	0.8	7	4 to 7
5. I will recommend this device to my friends and family.	6.5	0.8	7	4 to 7
6. Overall I am satisfied with the interface of the game.	6.5	0.6	7	5 to 7
7. Overall I am satisfied with the whole system.	6.5	0.7	7	5 to 7

Adverse or serious adverse events were assessed by querying participants after each session of BCI training if they have experienced any discomfort during the session. A summary of these events, if any, were then collated for each participant at the end of the study.

The efficacy outcome measure was the total scale index score of RBANS. RBANS is a brief clinical neuropsychological testing battery that was developed specifically for detecting and characterizing dementia in the elderly [27]. The battery comprises 12 subtests that assess the domains of Immediate and Delayed Memory, Language, Attention and Visuosptial/Construction [27]. It has four versions with two versions validated to have similar difficulty levels. Normative studies of the cross-cultural applicability of the RBANS in this elderly Chinese population have also been previously published [28]. Taking about 30 minutes to administer, its brevity, ease of use and sensitivity make it a useful instrument for evaluating elderly patients with abnormal cognitive decline.

Different versions of RBANS were counterbalanced and administered at different time points to counter practice effects. This assessment was administered by research assistants trained in the field of Psychology who are experienced in administering neuropsychological tests. These research assistants were not blinded to the study as it was deemed unfeasible for a small pilot trial. RBANS is an objective and standardized test. Scoring on each items are done according to objective scoring procedures detailed in the manual and requires little interpretation of the raw scores [27].

The primary endpoints were: safety and acceptability rate of the BCI device based on participants overall rating scores on a usability questionnaire; and change in RBANS total scale index score at week 8 from week 1. Acceptability rate was defined as proportion of participants whose rating score to the whole system was greater than 4 (scale range 1–7).

Secondary endpoints included: adherence rate which was defined as the proportion of participants who finished no fewer than 19 BCI sessions (out of 24 offered); changes in the five domain scores of the RBANS at week 8 from week 1, and the change between pre and post BCI sessions for the five domain scores of the RBANS and the total scale index score pooled across groups.

### Statistical Considerations

A total sample size of 32 participants was required to give a precision (width of 95% confidence interval) of approximately ±13% in the estimation of the proportion of participants who gave positive feedback on acceptability, assuming the true proportion was approximately 80%. In addition, we simultaneously evaluated the efficacy of the BCI system at week 8 for a decision of whether to proceed with a larger scale trial. For this purpose, Simon’s randomized phase II trial design was used, and a total sample size of 32 participants was determined so as to guarantee an 80% probability of correctly selecting the BCI arm as superior to the control if it was truly superior to the control group by an effect size of 0.3 [24], [29]. This design is used to select one of two arms as being worthy of further evaluation in a subsequent study but not to confirm the superiority of the selected arm.

All statistical significance tests and confidence intervals were two-sided. A p-value of <0.05 was considered statistically significant. All confidence intervals (CI) were calculated at the 95% level. All statistical analyses were conducted using SAS version 9.3 (Statistical Analysis System software, SAS Institute, North Carolina, USA). The changes in the RBANS scores at week 8 from week 1 were summarized and compared between the two groups using the Mann-Whitney U test. The median difference of the changes in the RBANS total scale index score between the two groups and its associated Hodges-Lehmann confidence interval were also estimated. The changes between pre and post BCI sessions for the domain scores and the total scale index score were pooled across groups and tested using the Wilcoxon-signed rank test.

No adjustments for multiplicity were conducted for the multiple tests in comparisons of RBANS scores, due to the explorative nature of this study.

## Results

### Participants

A total of 38 subjects were assessed for eligibility, among which 3 were excluded for not meeting the criteria. A total of 35 participants were randomized (17 in BCI intervention and 18 in waitlist control). Two participants were found to be ineligible and had been incorrectly randomized; neither received any of the intervention. This left a total of 33 patients, 15 in BCI intervention and 18 in waitlist control. One participant was lost to follow-up after randomization and one other withdrew after completing the week one assessment (both from the waitlist control arm). Neither received any intervention nor contributed any information beyond randomization or the baseline assessment (week one). Therefore, 31 (15 in BCI intervention and 16 in waitlist control) contributed information to the primary efficacy and acceptability analysis.

The mean age of participants was 65.1 (*SD* = 2.9) years and 60% were female. The majority of participants were educated to secondary level or below (57.1%) and self-reported to be familiar with computers (80.0%). The mean GDS score was 0.8 (*SD* = 1.1), with participants in the intervention arm showing slightly higher scores compared to the participants in the waitlist control arm, 1.1 (*SD* = 1.2) compared to 0.6 (*SD* = 1.0). The mean MMSE total score was 28.3 (*SD* = 1.3), with mean scores similar across arms.

### Primary Outcome Measures

#### Usability and acceptability

The mean and median responses of participants to the usability and acceptability questionnaire are presented in Table 1. All participants gave overall satisfaction rating as 4 or above (scale range from 1 “Strongly Disagree” to 7 “Strongly Agree”; 95% CI 89 to 100%). The mean scores for all items in the usability question were above 6.4 (Table 1).

#### Safety

There were no adverse or serious adverse events reported during the study period by any of the participants.

#### Changes in RBANS scores

**Figure 3:**
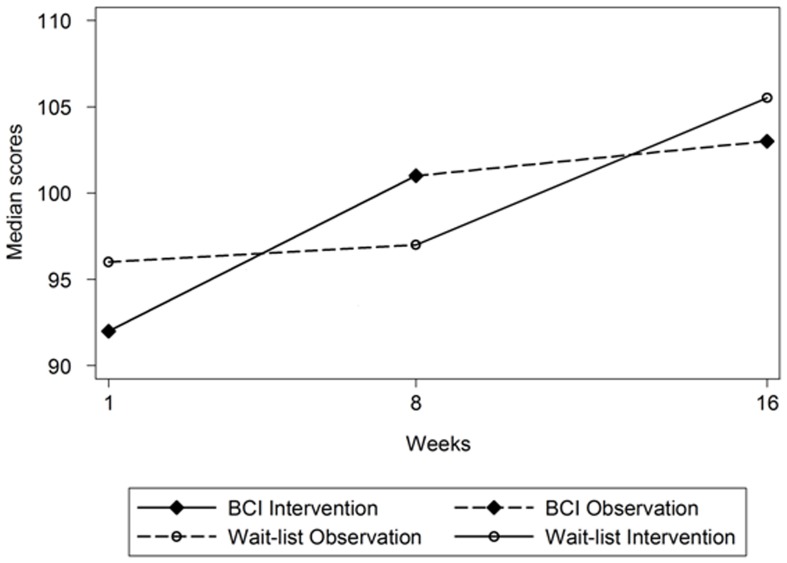
Plot of observed RBANS median total score over time by treatment arm.

The median scores of the two arms in the two periods are shown in Figure 3. The median of the difference in the RBANS total scale index score between week 8 and week 1 in the intervention arm pre and post training was 3.0 (range −6 to 28), as shown in Table 2. In the waitlist control arm, the corresponding median of the difference was 2.0 (−18 to 19) during the waitlist period. The waitlist control arm received the BCI intervention between week 9 and 16. The median of the difference in the RBANS total scale index score was 4.5 (−9 to 22) during the intervention period. The corresponding median of the difference between week 8 and week 16 in intervention arm who did not receive treatment during this period was 1.0 (−20 to 29).

**Table 2:** A comparison of change in RBANS Domain Index Scores between Week 1 and Week 8 for Intervention and Waitlist control arms.

Change in RBANS Scores between Week 1 and 8	Intervention	Wait-list	P-value^1^	Median diff. (95% CI)^2^		
**RBANS Domain Index Scores**						
**Immediate Memory**						
Mean (SD)	8.3 (18.4)	−1.8 (17.2)				
Median (range)	6.0 (−17 to 44)	−3.0 (−33 to 40)	0.160		9.5 (−3.0, 25.0)	
**Visuospatial/Constructional**						
Mean (SD)	4.1 (12.3)	3.5 (15.4)				
Median (range)	4.0 (−13 to 32)	1.5 (−21 to 37)	0.782		2.0 (−10.0, 12.0)	
**Language**						
Mean (SD)	0.1 (21.6)	−1.4 (20.7)				
Median (range)	−4.0 (−30 to 42)	0.0 (−36 to 38)	0.937		0.5 (−15.0, 17.0)	
**Attention**						
Mean (SD)	4.1 (12.2)	3.0 (13.5)				
Median (range)	6.0 (−27 to 25)	1.5 (−29 to 31)	0.677		1.0 (−6.0, 10.0)	
**Delayed Memory**						
Mean (SD)	6.5 (11.2)	2.1 (11.3)				
Median (range)	4.0 (−7 to 37)	0.0 (−24 to 22)	0.362		2.5 (−4.0, 11.0)	
**RBANS Total Scale Index Score**						
Mean (SD)	7.6 (11.4)	1.2 (11.3)				
Median (range)	3.00 (−6 to 28)	2.0 (−18 to 19)	0.332		7.0 (−4.0, 15.0)	

1 P-value from the Mann-Whitney U test.

2 Hodges-Lehmann estimation and its associated 95% confidence interval.

The Hodges-Lehmann estimate of the median difference in the change scores (from week 1 to week 8) of the total RBANS total scale index score between arms was 7.0 (95% CI: −4.0 to 15.0; p = 0.332) which was not statistically significant. The point estimate (7.0) reflects an effect size of approximately 0.6 SD. The Hodges-Lehmann estimate of the median differences between arms in the change scores (from week 1 to week 8) of the 5 RBANS domains ranged from 0.5 to 9.5, suggesting an improvement in each of the domains for those participants in the intervention arm. However, none of the differences in change scores across the domain scores were statistically significantly different (Table 2).

### Secondary Outcome Measures

#### Pooled analysis

Pooling the pre and post BCI data from both arms, the median of the changes in total score of the RBANS pre and post BCI was 4.0 (95% CI: −9.0 to 28.0; *p*<0.001) (Table 3).The median of the changes in immediate memory, visuospatial/constructional, attention and delayed memory domain scores pre and post BCI, both arms pooled, were all statistically significant, except the language domain score.

**Table 3:** Changes of RBANS individual index sub-scores and total scale index score pre and post intervention, pooling data from both Intervention and Waitlist control arms.

Change in RBANS scores pre and post-treatment	Summary statistics	P-value^1^
**RBANS Domain Index Scores**		
**Immediate Memory**		
Mean (SD)	6.9 (17.6)	
Median (range)	6.0 (−28 to 44)	0.038
**Visuospatial/Constructional**		
Mean (SD)	5.2 (11.2)	
Median (range)	4.0 (−13 to 32)	0.014
**Language**		
Mean (SD)	2.4 (16.8)	
Median (range)	0.0 (−30 to 42)	0.547
**Attention**		
Mean (SD)	3.4 (11.0)	
Median (range)	6.0 (−27 to 25)	0.039
**Delayed Memory**		
Mean (SD)	6.1 (10.0)	
Median (range)	6.0 (−12 to 37)	<0.001
**RBANS Total Scale Index Score**		
Mean (SD)	7.7 (10.1)	
Median (range)	4.0 (−9 to 28)	<0.001

1 P-value from the Wilcoxon signed rank test.

#### Adherence rate

All 31 participants completed all 24 sessions (adherence rate: 100%).

## Discussion

As seen from the responses for the usability and acceptability questionnaire, feedback from participants was positive. The very high adherence rate also suggested a high level of motivation among our participants. These factors indicated that elderly users may be sufficiently motivated to adhere to the training program even in their own homes.

RBANS total scale index scores improved by a similar magnitude pre and post training, and this occurred for both intervention and waitlist control arms (between Week 1 and 8 for intervention, and Week 9 and 16 for waitlist control). These scores did not change in the waitlist control arm before the intervention. In addition, between Weeks 9 and 16 when the intervention arm ceased treatment, their mean RBANS total scale index score neither decreased to baseline level nor improved at an equally large magnitude as between weeks 1 and 8. The time sequence of the changes can be taken as support that improvements in mean RBANS total scale index scores between weeks 1 and 8 for the intervention arm were due to our BCI treatment effect.

While the data indicated that the intervention arm showed a larger improvement in RBANS total scale index scores between Weeks 8 and 1 as compared to the waitlist control arm, this difference did not reach statistical significance. Thus, the study does not provide conclusive evidence for a difference in attention and memory in the normal elderly as assessed by the total scale index score on the RBANS between intervention arm and waitlist control. Nevertheless, an effect size of 0.6 SD was obtained. This is a moderate level of treatment effect according to Cohen [30]. According to Simon’s randomized phase II design, which aims to make a decision on whether to proceed to phase III trial or not, it can be concluded the intervention deserves further evaluation in a larger study [24], [29]. This decision is also supported by the highly significant result obtained when data from both arms were pooled, showing a positive shift in RBANS total scale index scores pre and post-BCI training.

It is notable that, pooling both arms, the scores for all five RBANS domains showed statistically significant positive changes pre and post BCI, except for language. This differentiated improvement suggests that gains in RBANS scores are valid indications of the efficacy of our training program, which targets attention and memory but not language. The significantly positive change in Visuospatial/Constructional could be attributed to the visual and pictorial nature of our memory task. This may have honed our participants’ attentiveness to pictorial stimuli, which are used to assess the Visuospatial/Constructional domain in RBANS. In addition, while the nature and modality of tasks used in training and assessment were very different (e.g. visual memory of pictures vs auditory memory of word lists), putative improvements in memory and attention during training were translated to score increases in both the relevant domain and global scores for RBANS. This could be taken as further evidence that our training results in global rather than task-specific improvements in cognitive functioning.

This study has a few limitations. Firstly, our current sample is limited only to English-literate elderly. Local statistics have shown that only 38% of Singapore residents 65 years old or above are literate in English [31], which suggests that our participants might constitute a smaller subset of our local geriatric population. A follow-up study currently underway is to translate and test the feasibility and efficacy of our BCI training program in a Chinese-speaking elderly population. The relatively small sample size is not a limitation, as the purpose of ours and that of the Simon’s design is to determine whether the product deserves evaluation in a large scale phase III trial.

Secondly, we acknowledge that there may be concerns about practice effects regarding the use of RBANS as an outcome measure for three time-points in close proximity. However, RBANS is one of the few neuropsychological tests for elderly that has alternate forms available; to counter practice effects, the four alternate forms were counterbalanced and administered at different time points such that no form was repeated for any single participant. In addition, no practice effect has been found in a previous study which examined the use of alternate forms at the time points [32].

The relatively small sample size is not a limitation, as our purpose in using the Simon’s design is to determine whether the product deserves evaluation in a large scale phase III trial. Definitive evidence about the efficacy of our intervention thus awaits a larger trial.

For this upcoming larger trial, we also plan to make our study design more rigorous by blinding the administration of RBANS and introducing a sham arm. These were not incorporated into the design of the current study as its primary purpose was to examine the safety, usability and acceptability of the device to our elderly participants. In addition, the absence of these features was not expected to have a substantial impact on the results of the current study for the following reasons. Firstly, RBANS is a standardized, manualized test battery that provides standard instructions to the participants and clear scoring guidelines, leaving little room for clinical judgment or potential subjective bias in administration and scoring [27]. As for a sham arm which may aid in distinguishing true intervention effects from a potential placebo effect, it was felt that objective tests of cognitive abilities such as list learning and digit span would not be particularly susceptible to possible influences of a placebo effect. Nevertheless, the inclusion of these features in our upcoming large trial may help to assuage any concerns in these areas that interested readers might have.

In conclusion, our BCI-based intervention shows promise in improving memory and attention in healthy elderly. A phase III trial is warranted and would potentially include participants who have mild cognitive impairment and early dementia. If shown to be efficacious in a larger trial, it may potentially serve as a novel intervention for reducing or even preventing cognitive decline in mildly cognitive impaired or Alzheimer’s disease patients.

## Supporting Information

Checklist S1 CONSORT Checklist.
(DOC)

Protocol S1 Trial Protocol.
(DOC)
