# Supplementary material for: A Brain-Computer Interface Based Cognitive Training System for Healthy Elderly: A Randomized Control Pilot Study for Usability and Preliminary Efficacy
Source: PLoS One. 2013 Nov 18;8(11):e79419. doi: 10.1371/journal.pone.0079419 (PMC3832588; doi:10.1371/journal.pone.0079419)
Supplement: Protocol S1 — Trial Protocol. (DOC) [file pone.0079419.s002.doc]

**NUS Institutional Review Board (IRB)**


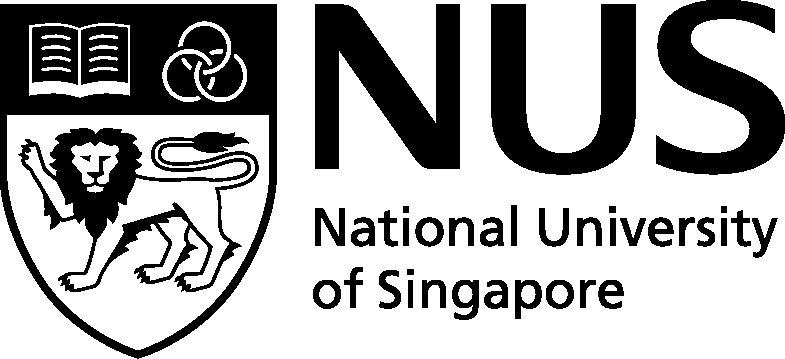
**APPLICATION FORM FOR SOCIAL,**

**BEHAVIOURAL & EDUCATIONAL RESEARCH**

| **I. BASIC INFORMATION** |
| --- |
| **Protocol (Research) Title:**  **Effectiveness of a Brain-Computer Interface Based System for Cognitive Enhancement in the Normal Elderly** |
| **Simplified Title (where applicable):**  (simplified title for Participant Information Sheet & Consent Form) |
| **Applicant:** |
| | Title | Name | Role | Position | Dept./Institution | | --- | --- | --- | --- | --- | | A/Prof | Lee Tih-Shih | PI / Supervisor  Corresponding PI | Associate Professor | Neuroscience and Behavioral Disorders |   *(please complete section III for all co-investigators)* |
| Source of Funding: |
| BMRC  NMRC  NUS ARF  Others  None  If Commercial Company/ Other organisation, please specify: Duke-NUS start up funds  Total amount of grant/fund: $ 150,000.00  Status of grant:  Approved  Pending  Not applicable |
| Nature of Research: |
| Archived/ Existing Database  Experiments  Questionnaire/ Survey  Others, please specify: |
| Research May Involve: |
| **Human Subjects**: (Target Number: 40)  Healthy Volunteers  Children (under 21 yrs old)  Pregnant Women  Outpatients  Inpatients  Prisoners  Cognitively Impaired Persons, please specify: ____________________ _________ |
| Research Subjects Will Be: |
| Reimbursed $400  Not reimbursed  Others – please specify: _________ |
| Has this research been rejected by any IRB / REC / DERCs? |
| No  Yes If yes, please provide details. |
| Site details: |
| Site(s) of research (Dept. & Institution): Duke-NUS Graduate Medical School and Training and Research Academy at Jurong Point (TaRA@JP)  Single-centre study  Multi-centre study - No. of local sites: 2 No. of overseas sites: |
| This research is also submitted to or has been approved by: |
| SingHealth:  SGH  NHC  NCC  CGH  SERI  KKH  NDC  NNI  SHP  NHG:  DSRB A  DSRB B  DSRB C  DSRB D  DSRB E  **Not Applicable** |

| **II. DECLARATION OF THE PRINCIPAL INVESTIGATOR** | |
| --- | --- |
| The information provided in this form is correct.   1. I will not initiate this research until I receive written notification of NUS-IRB approval and regulatory authority approval (if applicable). 2. I will not initiate any change in protocol without prior written approval from NUS-IRB except when it is necessary to reduce or eliminate risk to the subject. 3. I will promptly report any unexpected or serious adverse events, unanticipated problems or incidents that may occur in the course of this research. 4. I will maintain all relevant documents and recognize that the NUS-IRB staff and regulatory authorities may inspect these records. 5. I understand that failure to comply with all applicable regulations, institutional and NUS-IRB policies and requirements may result in the suspension or termination of this research. 6. I declare that there is no existing or potential conflict of interest for any of the investigators participating in this research.   Remarks (if any): | |
| ________________________________ | __________ |
| Principal Investigator’s signature | Date |
| Phone: Fax  Mailing Address: | |
| Email: | |

| **III. CO-INVESTIGATORS** |
| --- |
| *All co-investigators who have a responsibility for the consent process or direct data collection for this research should be listed below. Multiple copies of this form may be submitted as necessary. All co–investigators need not sign on the same form.* |
| Name: K Ranga Krishnan Email: [ranga.krishnan@duke-nus.edu.sg](mailto:ranga.krishnan@duke-nus.edu.sg)  Position: Dean, Professor Phone: 6516-7245  Department: Duke-NUS GMS Fax: 6226-3619  Institution: Duke-NUS Graduate Medical School  ___________________________ _________________________  Signature of Co-investigator Date |
| Name: Ng Tze Pin Email: [pcmngtp@nus.edu.sg](mailto:pcmngtp@nus.edu.sg)  Position: Associate Professor Phone: 6772-4514  Department: Psychological Medicine Fax: 6777-2191  Institution: National University of Singapore  ___________________________ _________________________  Signature of Co-investigator Date |
| Name: Guan Cuntai Email: [ctguan@i2r.a-star.edu.sg](mailto:ctguan@i2r.a-star.edu.sg)  Position: Programme Manager Phone: 6408-2663  Department: AIM Programme Fax: 6776-1378  Institution: Institute for Infocomm Research, A*STAR  ___________________________ _________________________  Signature of Co-investigator Date |
| Name: Feng Lei Email: [lei_feng@nuhs.edu.sg](mailto:lei_feng@nuhs.edu.sg)  Position: Research Fellow Phone: 6772-3491  Department: Psychological Medicine Fax:  Institution: National University of Singapore  ___________________________ _________________________  Signature of Co-investigator Date |
| Name: Cheung Yin Bun Email: [yinbun.cheung@scri.edu.sg](mailto:yinbun.cheung@scri.edu.sg)  Position: Head Phone: 6508-8310  Department: Biostatistics Fax: 6508-8317  Institution: Singapore Clinical Research Institute  ___________________________ _________________________  Signature of Co-investigator Date |
| Name: Donald Yeo Email: [donald.yeo.h.h@sgh.com.sg](mailto:donald.yeo.h.h@sgh.com.sg)  Position: Senior Clinical Neuropsychologist Phone: 6326-5355  Department: Neurology Fax: 6220 3321  Institution: Singapore General Hospital  ___________________________ _________________________  Signature of Co-investigator Date |

| **IV. COMMENTS OF HEAD OF DEPARTMENT *** *(Please circle accordingly)* | | |
| --- | --- | --- |
| **1. Significance:** |  |  |
| *Does the research address an important problem? Will the research affect concepts and methods that drive the field?* | **YES** | **NO** |
| **2. Approach:** |  |  |
| *Is the conceptual framework adequately developed? Are the design, methods and analyses adequately developed and appropriate?* | **YES** | **NO** |
| **3. Innovation:** |  |  |
| *Does the research challenge existing paradigms? Does it employ novel concepts, approaches and methods?* | **YES** | **NO** |
| **4. Principal Investigator:** |  |  |
| *Is the Principal Investigator appropriately trained to conduct this research? Does the Principal Investigator have evidence of commitment (e.g. previous track record)?* | **YES** | **NO** |
| **5. Environment:** |  |  |
| *Is the Principal Investigator’s environment suited to perform the research? Is there an adequate patient/subject pool and are there adequate resources?* | **YES** | **NO** |
| **6. Peer/ Scholarly/Scientific Review:** |  |  |
| *Has this project undergone a peer/ scholarly/ scientific review?* | **YES** | **NO** |
| **7. Budget (*to be completed ONLY for funded projects*):** |  |  |
| *If this research is funded, are the projected costs appropriate (i.e. accurate)?* | **YES** | **NO** |

Comments

I acknowledge that this research is in keeping with standards set by the Principal Investigator’s department.

| _________________________________________________ | ________ |
| --- | --- |
| Signature of Head / Chief / Vice-Dean(Research)* | Date |
| Name: Barry Halliwell |  |
| Title: Professor | Position: Deputy President of Research & Technology |

*The Department Representative can be the Head / Chief / Research Head of the PI’s Department.

If the PI or co-investigators is the Head or Chief of PI’s Department, this section should be completed by the Vice-Dean (Research) or Dean of the Faculty.

| **** IMPORTANT - Please complete Section V and VI, only if your existing research protocol submitted do not contain the relevant sections.** |
| --- |
| **V. ABSTRACT OF RESEARCH PROPOSAL** |
| *In no more than 300 words, describe concisely the specific aims, hypotheses, methodology and approach of the application, indicating where appropriate the application’s importance to science, existing knowledge and applications. The abstract must be self-contained so that it can serve as a succinct and accurate description of the application when separated from it. Please use lay terms. If this is not possible, the technical terms should be explained in simple language.* |
| The world population has reached an unprecedented seven billion, with global population ageing increasing at a greater rate than total population growth. Between 1998 and 2030, the proportion of persons aged 65 years and over in Singapore will grow by about 3% annually compared to 1.0-1.3% in some developed nations. Specific cognitive deficits like inattention, dysexecutive functioning, and processing speed decline may affect a number of quality of life domains. Concurrent with these statistics, the maintenance of the highest possible level of cognitive functioning for as long as possible has become an important goal of aging successfully.  To contribute to the realization of this goal we propose to conduct a wait-list control pilot trial to examine the efficacy and safety of a locally developed brain-computer interface based system (Brainpal^TM^) for cognitive enhancement in the normal elderly. Brainpal^TM^ uses a technology which analyzes brain waves captured through an electroencephalogram to determine the participants’ state of attention. The training program developed using this patented technology may be useful for individuals who experience difficulty with memory and sustaining their attention.  In the trial, we will randomize normal elderly aged 60 to 70 into two groups: they will receive the intervention at study onset or after 8 weeks of waiting. The primary objective is to examine the efficacy of 8-weeks of Brainpal^TM^ intervention for improving attention and memory in normal elderly. We hypothesize that elderly who have completed the training program will have significant improvement in their attention and memory compared to the controls, based on the Repeatable Battery for the Assessment of Neuropsychological Status. Brainpal^TM^ may represent one alternative means to enhance cognitive abilities and to slow down cognitive decline in the normal elderly. If demonstrated to be efficacious, this therapy may even help to delay the onset of dementia. |

| **VI. PROTOCOL CHECKLIST** |
| --- |
| Organise details of the research protocol under the following headings (in no more than 7 pages). |
| **1. Specific Aims:** |
| *1.1 State concisely and realistically what the research described in this application is intended to accomplish and/or what hypothesis is to be tested.*  This study aims to test the effectiveness of a novel Brain-Computer Interface (BCI) based system for cognitive enhancement in a normal elderly population. We hypothesize that normal elderly receiving treatment with the newly developed BCI-based training program will have memory and attention improvement to a greater extent as compared to a wait-list control group. |
| **2. Introduction:** |
| *2.1 Briefly describe the background to the current proposal.*  The world population has reached an unprecedented seven billion, with global population ageing increasing at a greater rate than total population growth (1). Between 1998 and 2030, the proportion of persons aged 65 years and over in Singapore will grow by about 3% annually compared to 1.0-1.3% in some developed nations (2). Cognitive impairment is the most consistent neurological impairment of acquired and degenerative brain disorders that comes with age. Specific cognitive deficits like inattention, executive dysfunction, and processing speed decline may affect a number of quality of life domains (3). Concurrent with these statistics, the maintenance of the highest possible level of cognitive functioning for as long as possible has become an important goal of aging successfully.    Cognitive training interventions have been found to improve targeted cognitive abilities in older adults (4). Despite the many mental training regimes that attempt to improve intelligence (e.g. 5), the mechanisms that underlie improvements in intelligence is not well understood (6). Recent studies combining neuroimaging and meditation support the notion that specific mental exercise will lead to selected changes in brain circuitry that can significantly affect information processing and behavior (7). This process-based training that targets specific cognitive processes require careful task analysis to determine what processes are being trained and to pick the appropriate transfer tasks (8).  Brain-computer Interface (BCI) is a direct communication pathway between a human brain and an external device. It is a technology that enables people to interact with computers through their thoughts. Electroencephalogram (EEG) is the best studied non-invasive interface facilitating such communication, mainly due to its fine temporal resolution, ease of use, portability and low set-up cost (9). It presents the user with real-time feedback on brainwave activity, as measured by electrodes on the scalp, typically in the form of a video display, sound or vibration (10). Traditional EEG biofeedback encourages peak alpha frequency (PAF) which has been shown to correlate positively with cognitive performance and correlate negatively with age after childhood (11). At a neuronal level, age-related cognitive decline involves prefrontal cortex (PFC) dysfunction (12).  Taking this information, we propose to conduct a pilot study to examine the efficacy of a locally developed BCI-based system (Brainpal^TM^) for cognitive enhancement in the normal elderly. The BCI system will take EEG recordings from the PFC to determine the participants’ state of attention with high specificity. The training program developed using this patented technology may be useful for individuals who experience difficulty with memory and sustaining their attention. Therefore, we aim to evaluate its efficacy and safety in improving memory and attention in normal elderly through a wait-list control pilot trial. |
| *2.2 State concisely the importance of the research described in this application.*  Therapy using non-invasive BCI system-based games may represent one alternative means to enhance cognitive abilities and to slow down cognitive decline in the normal elderly. If demonstrated to be efficacious, this therapy may even help to delay the onset of dementia. It is also likely to be preferred over other modalities of cognitive enhancement like drugs (13) which has potentially serious side effects and high risk of abuse. |
| *2.3 Relevant references*   1. Tollefson J. Seven billion and counting. Nature 2011; 478: 300. 2. United Nations Department of Economic and Social Affairs Population Division. World Population Ageing 2009. Updated 2010. 3. Mitchell AJ, Kemp S, Benito-Leon J, Reuber M. The influence of cognitive impairment on health-related quality of life in neurological disease. Acta Neuropsychiatrica 2010; 22: 2-13. 4. Ball K, Berch DB, Helmers KF, Jobe JB, Leveck MD, Marsiske M et al. Effects of Cognitive Training Interventions with Older Adults: A Randomized Controlled Trial. JAMA 2002; 288: 2271-2281. 5. Rasmusson DX, Rebok GW, Bylsma FW, Brandt J. Effects of Three Types of Memory Training in Normal Elderly. Aging, Neuropsychology, and Cognition 1999; 6: 56-66. 6. Buschkuebl M, Faeggi SM. Improving Intelligence: a literature review. Swiss Med Weekly 2010; 140: 266-272. 7. Slagter HA, Davidson RJ, Lutz A. Mental training as a tool in the neuroscientific study of brain and cognitive plasticity. Frontiers in Human Neuroscience 2011; 5: 1-12. 8. Lustig C, Shah P, Seidler R, Reuter-Lorenz PA. Aging, Training, and the Brain: A Review and Future Directions. Neuropyschol Review 2009; 19: 504-522. 9. Lebedev MA, Nicolelis MAL. Brain-Machine Interfaces: past, present, and future. Trends in Neuroscience 2006; 29: 536-546. 10. Hirshberg LM, Chiu S, Frazier JA. Emerging brain-based interventions for children and adolescents: overview and clinical perspective. Child Adolesc Psychiatric Clin N Am 2005; 14: 1-19. 11. Angelakis E, Stathopoulou S, Frymiare JL, Green DL, Lubar JF, Kuonios J. EEG Neurofeedback: A brief overview and an example of peak alpha frequency training for cognitive enhancement in the elderly. The Clinical Neuropsychologist 2007; 21: 110-129. 12. Wang M, Gamo NJ, Yang Y, Jin LE, Wang XJ, Laubach M et al. Neuronal basis of age-related working memory decline. Nature 2011; 476: 210-213. 13. Greely H, Sahakian B, Harris J, Kessler RC, Gazzaniga M, Campbell P et al. Towards responsible use of cognitive-enhancing drugs by the healthy. Nature 2008; 456: 702-705. |
| **3. Preliminary Studies:** |
| *3.1 Provide an account of the Principal Investigator’s preliminary/pilot studies (if any) pertinent to the application.*  We conducted a pilot non-randomized trial to test the Brainpal^TM^ treatment protocol and its efficacy on 20 children with ADHD. 10 ADHD children went through 10 weeks of the Brainpal^TM^ intervention, while another 10 children with ADHD served as controls. The 20 session intervention program was well accepted by the parents and children. The ADHD rating scales administered to parents and teachers both showed good test-retest reliability (>0.9). After 20 sessions of therapy, having adjust for baseline score, children in the intervention group showed statistically significant difference in the attention problem score on the ADHD Rating Scale at 10 weeks compared to children in the control group, with a mean (SD) reduction of inattentive scores of 2.9 (4.0) for the treatment versus -0.5 (2.0) for the control group (p<0.05). Results from this pilot study were published in Psychopharmacology Bulletin 2010; 43: 73-82. |
| **4. Methodology:** |
| - 1. *Discuss in detail the design and procedures to be used to accomplish the specific aims of the research.*   The study will take place over the course of 16 weeks. An intake assessment will be done at the pre- screen and the participant should fulfill the following criteria:   1. Age range between 60-70 years old 2. Clinical Dementia Rating (CDR) of 0 3. Geriatric Depression Scale (GDS) of 9 and below 4. Mini-Mental State Examination of 26 and above 5. Chinese ethnicity 6. Literate in English 7. Able to travel to study site independently 8. Retired and/or not currently employed   They should also not have any of the exclusion criteria listed below:   1. Any known neuropsychiatric disorders (such as epilepsy or mental retardation) 2. Involvement in another research study (aside from SLAS)   Informed consent will be obtained from the participant prior to any form of assessment or intervention done as part of the study. The details of the study will be explained to the participants and they will be allowed sufficient time to think about the study and voice any queries pertaining to the informed consent. Participants who express an interest in taking part will be scheduled for a pre-screen appointment.  DESIGN  40 participants will be recruited for this study. There will be an intervention group and a waitlist control group to observe the effects of the BCI treatment on the elderly population. Subjects will be randomized into either the intervention or waitlist control group, with 20 participants in each group. Subjects in both the intervention and waitlist control group will undergo the BCI intervention for 24 sessions over the span of 8 weeks. Each session will take 30-minute to complete. The intervention group will undergo the BCI treatment in the first 8 weeks of the trial and the waitlist control will start their 8 week treatment after the completion of the intervention group from week 9 onwards.  The primary outcome measure of the study is the change in the total score of the Repeatable Battery for the Assessment of Neuropsychological Status (RBANS). The secondary outcome measure is the change in the index and subtest scores on the RBANS and scores of the Conner’s Performance Test (CPT). |
| - 1. *Include details on sample size calculation and the means by which data will be analysed and interpreted.*   Sample size plan  Assuming a moderate effect size of 0.4SD on RBANS total score improvement, power of 80% and type I error of 5%, a sample size of approximately 20 participants per group is necessary. This includes an allowance for up to 20% losses to follow-up. The assumed effect size of 0.4SD is conservative in comparison with the effect size of 0.9SD in the pilot study (albeit a different scale), and is considered small-to-medium according to Cohen.  Statistical analysis  Groups will be compared for balance in baseline characteristics and drop-out rate in CONSORT flow chart. Efficacy analysis will be on intention-to-treat basis. The primary outcome will be improvement on the RBANS total scores at 8 weeks. The main analysis will be based on two-sample t-test. Sensitivity analysis by Mann-Whitney U test will be performed. 95% confidence interval for the treatment effect will be presented.  Differences in secondary endpoints at 8 weeks between groups will be assessed by two-sample t-test and Fisher’s exact test for continuous and categorical outcomes respectively. As the main study conclusion is based on a single primary endpoint and the secondary endpoints are only for further understanding, there will be no adjustment for multiplicity.  Booster effect will be analyzed by first pooling paired data before and after the boosters in the two groups. Pre and post booster comparisons will be made in the above pooled data by using the paired t-test, with Wilcoxon signed rank test as sensitivity analysis. |
| - 1. *List all subject-related procedures. Please also describe the subject research visits (frequency and procedures involved).For studies with multiple visits, please attach visits schedule.*   INTERVENTION GROUP  The 20 participants in this group will undergo a series of 24 training sessions over the span of 8 weeks (3 visits per week). During the first session, the participants will have to do a calibration task (Stroop task) in order for the system to recognize the baseline level of concentration for each participant. After this, the participant will then go on to take part in a series of training activity. Each training activity employs the BCI system, and controlled by the subject’s concentration, is fed to the computer via the EEG leads. The participant will have to use their concentration in order to maneuver a series of tasks on the computer screen. At the end of the 24 sessions, they will do another Stroop task in order to assess whether there has been a change in their baseline level of concentration. The RBANS and CPT will be administered in their baseline session, last session and at the end of the 16-week trial.  WAITLIST CONTROL GROUP  The remaining 20 participants complete a calibration task at week 1. After which, they will not receive any BCI training in the first 8 weeks of the trial. They begin their BCI training at week 9 and undergo the same procedures as the intervention group. The RBANS and CPT will be administered in their baseline session, last session and at the end of the 16-week trial.   |  | | Pre-Study  Screening | Baseline (Intervention) | Treatment Visits | Post-treatment (Intervention)  Baseline  (Waitlist control) | Treatment Visits | Follow-up (Intervention) Post-treatment  (Waitlist control) | | --- | --- | --- | --- | --- | --- | --- | --- | | Time point | | T0 | T1 |  | T2 |  | T3 | | Week | | Week 0 | Week 1 | Week 1-8 | Week 8 | Week 8-16 | Week 16 | | Informed Consent | Both | XX |  |  |  |  |  | | CDR Scale | Both | XX |  |  |  |  |  | | GDS | Both | XX |  |  |  |  |  | | MMSE | Both | XX |  |  |  |  |  | | BCI Training | Intervention |  | X | X | X |  | X | | Waitlist control |  | X |  | X | X | X | | RBANS | Intervention |  | X |  | X |  | X | | Waitlist control |  | X |  | X |  | X | | CPT | Intervention |  | X |  | X |  | X | | Waitlist control |  | X |  | X |  | X | |
| - 1. *What are the anticipated benefits and risks to human subjects participating in this research?*   Subjects who complete the 8 weeks of intensive Brainpal^TM^ training may have improved memory and attention. We do not expect any risks to the subjects. |
| - 1. *Discuss the potential difficulties and limitations of the proposed procedures and alternative approaches to achieve the aims. Please provide information on how these limitations/ difficulties faced may be minimized or overcome.*   Subjects may have difficulty making frequent visits to the study site for the Brainpal^TM^ intervention sessions. Thus, we will only recruit those who have retired and are able to travel independently to the study site for this study. |
| - 1. *Will any part of the procedures be placed on audiotape, film/video, or other electronic media?*  *Yes*  *No*   *If Yes, what is the media? Explain how the recorded information will be used? How long will the tapes, etc, be retained and how will they be disposed of?* |
| **5. Additional Information on Methodology:** |
| *5.1 If research involves creating new databases or making use of archived/ existing databases, please complete the sections below. In addition, if your research involves making use of archived/ existing databases, please furnish the necessary documentation, e.g., permissions to use those databases.*   - - 1. Where will the data be stored?   Data will be stored without personal identifiers in the password protected computers of the PI and Co-PI. Hardcopies of the data will be filed and locked in a cabinet.   - - 1. Who will have access to the data?   Only the PI, Co-PI and designated research associates and collaborators will have access to the data.   - - 1. What will happen to the data after research completion?   Study data will be stored in a lock cabinet for 5 years, after which hardcopies will be destroyed.   - - 1. Any other remarks? |
| **6. Characteristics of Target subjects / Target Subject Data:** |
| - 1. What is the number of subjects to be enrolled? Give a breakdown by institution for multi-center studies (if applicable).  | Institution/Site of Recruitme | t  Total | Men | Women | Children | | --- | --- | --- | --- | --- | |  | 40 | 20 | 20 | 0 |  - 1. Lower Age Limit: 60 Upper Age Limit (if any): 70   2. Total number of subjects targeted for enrolment worldwide (for international studies): NA   3. Are there any subject recruitment restrictions based on race of the subject?   Yes, we will only recruit Chinese subjects.   - 1. Inclusion criteria  1. Age range between 60-70 years old 2. Clinical Dementia Rating (CDR) of 0 3. Geriatric Depression Scale (GDS) of 9 and below 4. Mini-Mental State Examination of 26 and above 5. Chinese ethnicity 6. Literate in English 7. Able to travel to study site independently 8. Retired and/or not currently employed    1. Exclusion criteria 9. Any known neuropsychiatric disorders (such as epilepsy or mental retardation) 10. Involvement in another research study (aside from SLAS)     1. Are the subjects vulnerable or in a dependent relationship with the researchers?   Yes  No  If Yes, please provide details. |
| **7. Participant Information Sheet and Written Informed Consent Form:** |
| - 1. *The PI is responsible for ensuring that all research subjects give informed consent before enrolling into the research. Please submit a copy of the Participant Information Sheet and Consent Form.*   *(A sample of Participant Information Sheet and Consent Form is available on the IRB website at* [*http://www.nus.edu.sg/irb/Guide.htm*](http://www.nus.edu.sg/irb/Guide.htm)*)* |
| - 1. *Summarise the consent procedure. Please specify how will informed consent be obtained and who will obtain consent.*   The Participant Information Sheet will be explained to the subject by trained research assistants in English. The subject will sign on the consent form and informed consent will be obtained prior to any other study involvement activities. |
| - 1. ***If waiver of consent is required****, please justify how your research meets the following criteria:*   *(The NUS-IRB may waive the requirement to obtain informed consent if the NUS-IRB finds that the research meets the following 4 criteria. Please note that all studies involving* ***deception*** *must apply for waiver of informed consent. Subjects in these studies* ***must*** *be debriefed.)*   - - 1. *The research involves no more than minimal risk to the subjects.*      - - 1. *The waiver or alteration will not adversely affect the rights and welfare of the subjects.*      - - 1. *Whenever appropriate, the subjects will be provided with additional pertinent information after participation.*      - - 1. *The research could not practicably be carried out without the waiver or alteration.* |
| **8. Recruitment Process:** |
| - 1. *Explain the process of recruitment in detail. For example state how the list of potential research subjects will be obtained. Please submit a copy of any advertisements/posters that will be used.*   The study population will be primarily drawn from an existing large-scale longitudinal study of elders (SLAS) but the proposal provides for direct recruitment as well. Potential subjects from the SLAS study will be screened against the inclusion and exclusion criteria and subjects fulfilling all criteria will be invited to take part in this study.  For direct recruitment, the research team will identify relevant seminars and talks with potential participants and approach the organizer of the seminars to recruit during the event. Recruitment will be in the form of a short presentation explaining our study aims, procedures, inclusion and exclusion criteria required of our subjects. A copy of the slides to be used is attached in the email for NUS-IRB reference. |
| **9. Timelines:** |
| - 1. *What are the estimated start and end dates of the research?*   *Start Date:* January 2012 *End Date:* June 2013 |
| - 1. *Indicate the duration of subject involvement in the research. Please also state the recruitment period.*   Duration of each subject’s involvement: 16 weeks. Recruitment period: January to June 2012 or when IRB approval is obtained. |
| **10. Financial Aspects/Conflicts of Interest:** |
| - 1. *Who will be responsible for research related costs? For sponsored research, list the costs that will be borne by the sponsor.*   All research related costs for this study will be borne by the Duke-NUS Graduate Medical School start-up grant. |
| - 1. *For industry sponsored research, please complete the following.*      1. *Name of the sponsor company?*   NA   - - 1. *Address of the sponsor?*   NA   - - 1. *Sponsor contact person?*   NA   - - 1. *Have any of the investigators received any financial support, sponsorship from the company supporting this research?*  *Yes*  *No*     2. *Do any of the investigators hold any ownership interest, e.g. stock options in this company?*  *Yes*  *No*     3. *Is the sponsor offering any incentive connected with subject recruitment or completion of research (e.g. finders’ fee, recruitment bonuses etc) that will be paid to the research staff?*  *Yes*  *No*     4. *If you have answered ‘yes’ to Q 10.2.5, please elaborate.*      - - 1. *Any other remarks?* |
| - 1. *Will subjects receive financial payment/ incentive for participation? If yes, please elaborate.*   Yes, subjects will receive $40 for each visit that involves questionnaire assessments (i.e. at pre-screen, baseline, week 8, and week 16), and $10 for each BCI intervention visit. Thus, each subject who completes the study will receive ($40x4) + ($10x24) = $400 in total. The payment is to offset their travelling costs and to compensate their time. |

| **VII. ATTACHMENT CHECKLIST:** | | |
| --- | --- | --- |
| **Document** | **Attached?** | **Not Applicable?** |
| Research Protocol (latest version)*^+^ |  |  |
| Grant Application Form |  |  |
| Participant Information Sheet and Consent Form^+^ |  |  |
| Investigator(s)’ CV |  |  |
| Survey Form(s)/Questionnaire(s) / Interview Guide ^+^ |  |  |
| Data Collection Form^+^ |  |  |
| Advertisement for Recruitment of Subjects^+^ |  |  |
| Letter of Invitation to Subjects^+^ |  |  |
| Relevant Publications |  |  |
| Subject Payment Details* |  |  |
| Financial Agreement |  |  |

^+^Version number and date is required.

*If information is not included in sections V and VI of the application form.
